# BCL11A overexpression predicts survival and relapse in non-small cell lung cancer and is modulated by microRNA-30a and gene amplification

**DOI:** 10.1186/1476-4598-12-61

**Published:** 2013-06-12

**Authors:** Ben-yuan Jiang, Xu-chao Zhang, Jian Su, Wei Meng, Xue-ning Yang, Jin-ji Yang, Qing Zhou, Zhi-yong Chen, Zhi-hong Chen, Zhi Xie, Shi-liang Chen, Yi-long Wu

**Affiliations:** 1 Guangdong Lung Cancer Institute, 106 Zhongshan Er Rd, Guangzhou, 510080, China; 2 Medical Research Center of Guangdong General Hospital, Guangdong Academy of Medical Sciences, 106 Zhongshan Er Rd, Guangzhou, 510080, China; 3 Graduate School of Southern Medical University, Guangzhou, 510515, China

**Keywords:** BCL11A, Proto-oncogene, Non-small cell lung cancer, microRNA, Prognosis

## Abstract

**Background:**

Aberrant activation of the proto-oncogene B-cell lymphoma/leukemia 11A (*BCL11A*) has been implicated in the pathogenesis of leukemia and lymphoma. However, the clinical significance of BCL11A in non-small cell lung cancer (NSCLC) remains unknown.

**Results:**

We examined BCL11A expression at the protein and mRNA levels in a cohort (n = 114) of NSCLC patients and assessed the relationship between BCL11A expression and clinicopathological parameters. Data from array-based Comparative Genomic Hybridization (aCGH) and microRNA transfection experiments were integrated to explore the potential mechanisms of abnormal BCL11A activation in NSCLC. Compared to adjacent non-cancerous lung tissues, BCL11A expression levels were specifically upregulated in NSCLC tissues at both the mRNA (*t* = 9.81, *P <* 0.001) and protein levels. BCL11A protein levels were higher in patients with squamous histology (χ^2^ = 15.81, *P* = 0.001), smokers (χ^2^ = 8.92, *P* = 0.004), patients with no lymph node involvement (χ^2^ = 5.14, *P* = 0.029), and patients with early stage disease (χ^2^ = 3.91, *P* = 0.048). A multivariate analysis demonstrated that in early stage NSCLC (IA–IIB), BCL11A was not only an independent prognostic factor for disease-free survival (hazards ratio [HR] 0.24, 95% confidence interval [CI] 0.12-0.50, *P* < 0.001), but also for overall survival (HR = 0.23, 95% CI 0.09-0.61, *P* = 0.003). The average BCL11A expression level was much higher in SCC samples with amplifications than in those without amplifications (*t* = 3.30, *P* = 0.023). Assessing functionality via an *in vitro* luciferase reporter system and western blotting, we found that the BCL11A protein was a target of miR-30a.

**Conclusions:**

Our results demonstrated that proto-oncogene *BCL11A* activation induced by miR-30a and gene amplification may be a potential diagnostic and prognostic biomarker for effective management of this disease.

## Background

Lung cancer, especially non-small cell lung cancer (NSCLC), is the leading cause of cancer-associated deaths in China and worldwide [1]. Despite advances in early diagnosis and standard treatment, the prognosis of NSCLC patients remains poor, with 5-year survival less than 15% [2]. A better understanding of the molecular mechanisms underlying lung cancer is needed for more efficient cancer management [1,3]. Molecular epidemiological studies have provided evidence that multiple mechanisms, including activation of oncogenes (*EGFR*, *KRAS*), inactivation of tumor suppressors (*TP53*), and dysregulation of DNA repair genes (*CHEK1*), contribute to early stage lung cancer development [1,4,5]; however, these molecular alterations are not enough to explain the heterogeneity of NSCLC. Identification of relevant molecular alterations occurring at early stage disease has the potential to identify diagnostic or predictive biomarkers and may provide a strategy for clinically tailored treatments to decrease the overall mortality of this disease.

The B-cell lymphoma/leukemia 11A (*BCL11A*) gene encodes a Krüppel zinc-finger transcription factor, which has been shown to be essential for pre-B-cell development, thymocyte maturation, and globin switching [6,7]. This gene was first identified in a rare t (2;14) (p16;q32.3) translocation in aggressive B-cell chronic lymphocytic leukemia and was regarded as a candidate oncogene, as it was often co-amplified with the *REL* proto-oncogene in non-Hodgkin’s lymphoma and in classical Hodgkin’s lymphoma [8,9]. Furthermore, retroviral integration into the *BCL11A* locus has been shown to activate its expression, transform NIH 3T3 cells, and induce myeloid leukemia in mice [6,10].

BCL11A involvement in solid tumors has been rarely reported, and whether deregulation of BCL11A expression occurs in NSCLC remains unclear. To address BCL11A expression and its potential clinical relevance in detail, we determined BCL11A expression at both the mRNA and protein levels and determined its prognostic significance by correlating BCL11A expression with clinicopathologic features and survival in NSCLC patients. We also investigated the mechanisms underlying BCL11A activation in NSCLC.

## Results

### *BCL11A* mRNA is specifically upregulated in NSCLC tissues

**Figure 1:**
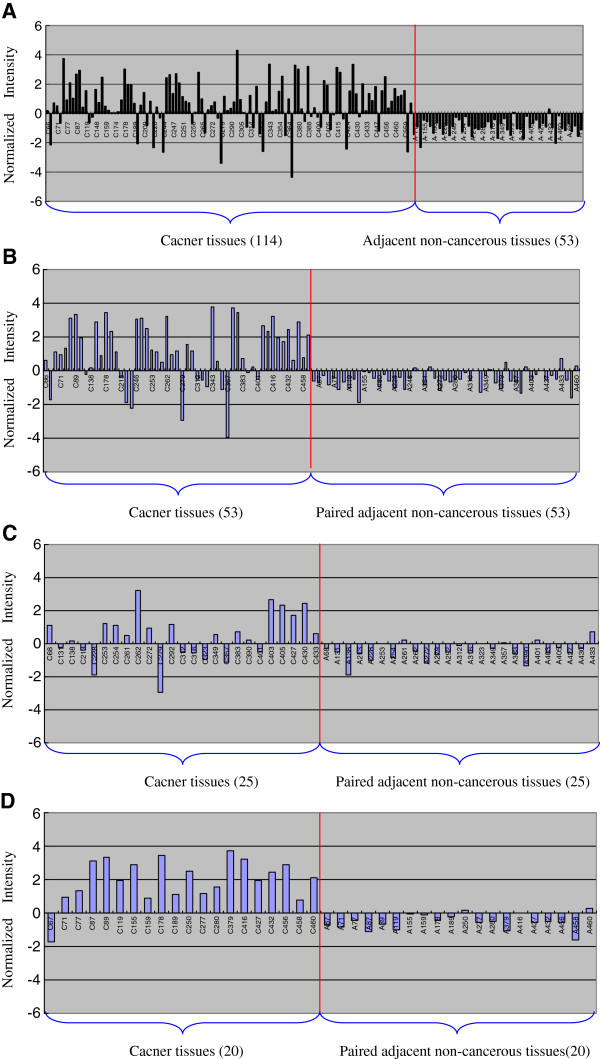
***BCL11A *mRNA is upregulated in cancer tissues compared to adjacent non-cancerous tissues.** Normalized intensity of *BCL11A* expression was determined by Affymetrix® mRNA array and compared between (**A**) all 114 NSCLC cancer tissues and 53 adjacent non-cancerous lung tissues; (**B**) 53 NSCLC cancer tissues and paired adjacent non-cancerous lung tissues; (**C**) 25 paired lung adenocarcinoma and adjacent lung tissues; (**D**) 20 paired lung squamous carcinoma and adjacent lung tissues. *BCL11A* was specifically upregulated in NSCLC tissues, especially in squamous cell lung carcinoma tissues.

Based on our gene expression profiling data, we found that *BCL11A* was differentially expressed between cancer and adjacent non-cancerous tissues, with 3.06-fold upregulation in cancer tissues (*t* = 9.81, *P <* 0.001). Whether comparing between all cancer and adjacent tissues or just between paired cancerous and non-cancerous tissues, *BCL11A* mRNA was upregulated in cancer tissues (Figure 1A, B). Furthermore, when *BCL11A* expression was analysed according to different histological subtypes, *BCL11A* mRNA was upregulated mainly in squamous cell carcinoma (SCC) (Figure 1C, D). A correlation analysis was performed to explore whether *BCL11A* mRNA levels were related to clinicopathological variables in patients with NSCLC. *BCL11A* mRNA levels had no relationship with age, sex, or histology, but they did correlate with lymph node status and disease stage. Decreased *BCL11A* mRNA levels were associated with lymph node involvement (χ^2^ = 6.17, *P* = 0.013) and advanced stage (χ^2^ = 4.21, *P* = 0.040) (Table 1).

**Table 1:** Relationships between BCL11A expression and clinicopathological factors.

	**BCL11A mRNA expression (n = 114)**				**BCL11A protein expression (n = 113)**			
**Parameter**	**No.**	**Low (%)**	**High (%)**	***P-*value**	**No.**	**Low (%)**	**High (%)**	***P*-value**
Age				0.984				0.325
<60 yr	50	21 (42.0)	29 (58.0)		70	44 (62.9)	26 (37.1)	
≥60 yr	64	27 (42.2)	37 (57.8)		43	23 (53.5)	20 (46.5)	
Sex				0.708				0.133
Female	33	13 (39.4)	20 (60.6)		36	25 (69.4)	11 (30.6)	
Male	81	35 (43.2)	46 (56.8)		77	42 (54.5)	35 (45.5)	
Performance status				0.826				0.399
0	58	25 (43.1)	33 (56.9)		56	31 (55.4)	25 (44.6)	
1–2	56	23 (41.1)	33 (58.9)		57	36 (63.2)	21 (36.8)	
Smoking status				0.617				0.003
No	53	21 (39.6)	32 (60.4)		56	41 (73.2)	15 (26.8)	
Yes	61	27 (44.3)	34 (55.7)		57	26 (45.6)	31 (54.4)	
Histological type				0.921				0.001
AC	68	29 (42.6)	39 (57.4)		68	50 (73.5)	18 (26.5)	
SCC	35	15 (42.9)	20 (57.1)		36	12 (33.3)	24 (66.7)	
LCC	11	4 (36.4)	7 (63.6)		9	5 (55.6)	4 (44.4)	
Tumor size				0.345				0.124
≤ 3.0 cm	63	29 (46.0)	34 (54.0)		59	39 (66.1)	20 (33.9)	
> 3.0 cm	51	19 (37.3)	32 (62.7)		54	28 (51.9)	26 (48.1)	
Lymph node status				0.013				0.023
N0	72	24 (33.3)	48 (66.7)		72	37 (51.4)	35 (48.6)	
N1–2	42	24 (57.1)	18 (42.9)		41	30 (73.2)	11 (26.8)	
Pathological stage				0.040				0.048
I–II	89	33 (37.1)	56 (62.9)		88	48 (54.5)	40 (45.5)	
III–IV	25	15 (60.0)	10 (40.0)		25	19 (76.0)	6 (34.0)	

### BCL11A protein is specifically upregulated in NSCLC tissues

**Figure 2:**
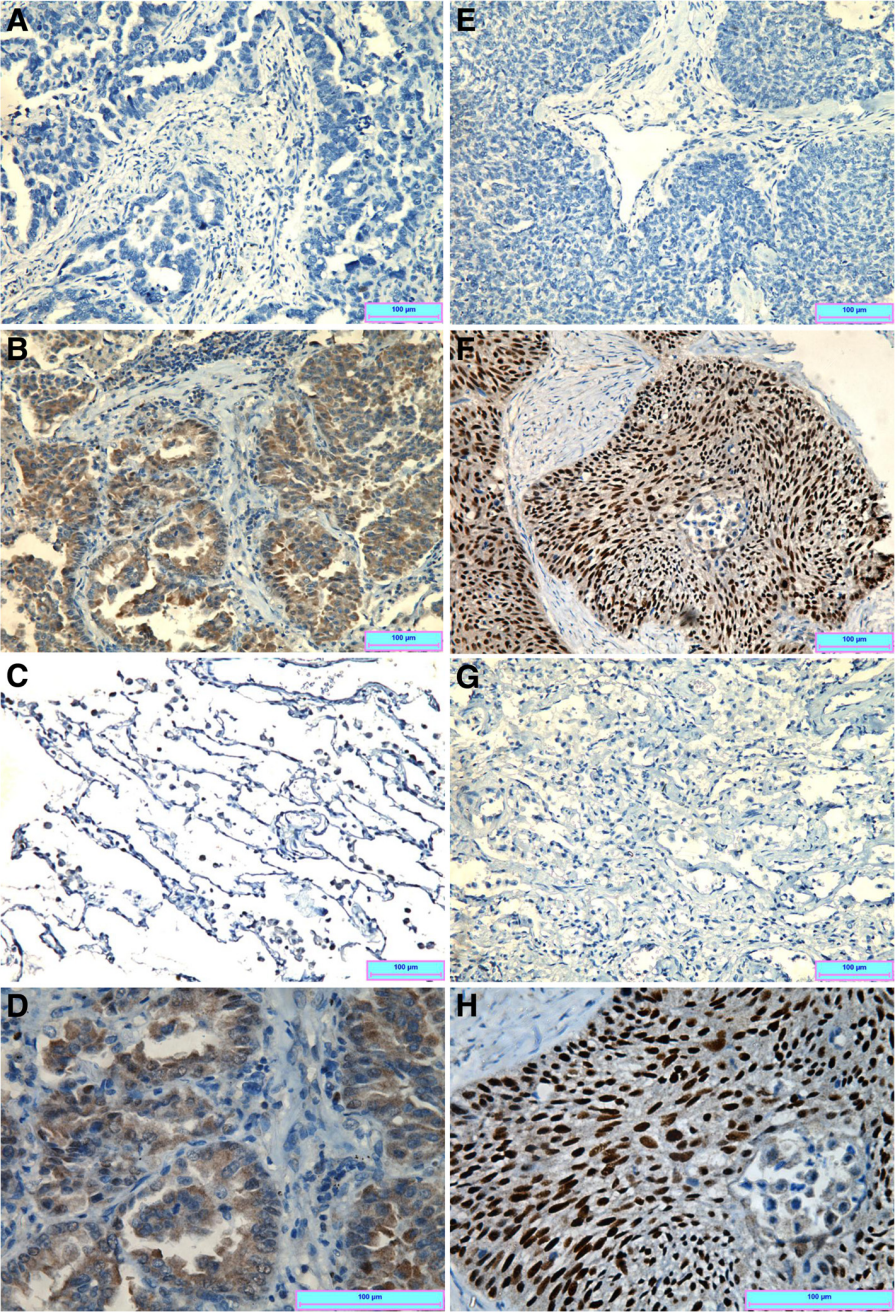
**Detection of BCL11A protein expression by immunohistochemical staining in cancer and adjacent tissues.** Representative images of (**A**) negative or (**B**) positive staining of BCL11A in adenocarcinoma cells and (**C**) negative staining in adjacent lung tissues. (**D**) A zoom-in view of BCL11A expression in lung adenocarcinoma cells. Similarly, representative images of (**E**) negative or (**F**) positive staining of BCL11A in lung squamous carcinoma cells and (**G**) negative staining in adjacent lung tissues. (**H**) A zoom-in view of BCL11A expression in lung squamous cell carcinoma cells.

Expression of BCL11A at the protein level was also investigated in 113 NSCLC and 25 adjacent non-cancerous lung tissues by immunohistochemistry. BCL11A protein staining was not found in the 25 adjacent lung tissues (Figure 2). Statistical analysis revealed no significant correlations between BCL11A protein level and age, sex, or performance status. However, BCL11A expression level was strongly associated with histology (χ^2^ = 15.81, *P* = 0.001), smoking status (χ^2^ = 8.92, *P* = 0.004), lymph node status (χ^2^ = 5.14, *P* = 0.029), and disease stage (χ^2^ = 3.91, *P* = 0.048) (Table 1).

### BCL11A protein level correlates with disease-free survival (DFS) and overall survival (OS) in early stage squamous carcinoma NSCLC patients

For all 113 patients subjected to immunohistochemical staining for BCL11A, 68 (59.2%) had low BCL11A expression and 45 (40.8%) had high BCL11A expression.

**Figure 3:**
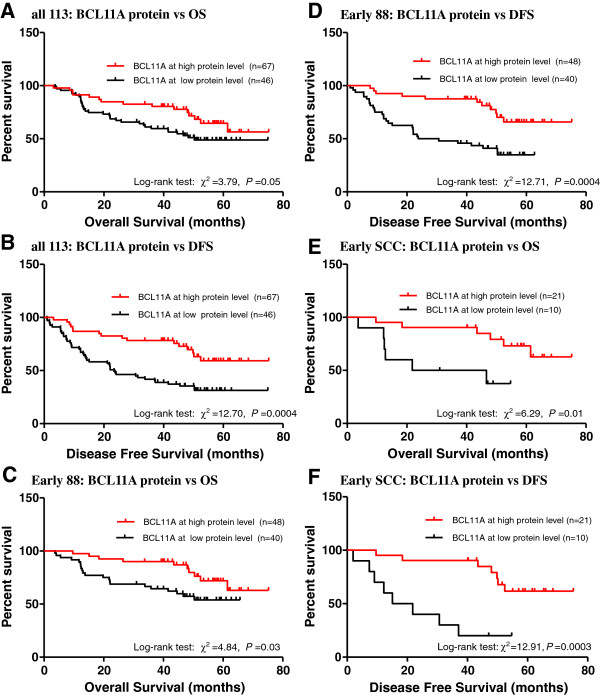
**Kaplan-Meier estimated overall survival and disease-free survival curves of NSCLC patients according to BCL11A protein level.** (**A**) Overall survival of all 113 NSCLC patients. (**B**) Disease-free survival of all 113 NSCLC patients. (**C**) Overall survival of 88 early stage NSCLC patients. (**D**) Disease-free survival of 88 early stage NSCLC patients. (**E**) Overall survival of 31 patients with early stage squamous cell lung cancer. (**F**) Disease-free survival of 31 early stage squamous cell lung cancer patients. *P* values were calculated by log rank tests.

High BCL11A expression was predictive of better overall survival (OS) (log rank χ^2^ = 3.79, *P* = 0.05) and better disease-free survival (DFS) (log rank χ^2^ = 12.70, *P* = 0.0004) (Figure 3A, B). The median DFS of patients with low BCL11A expression levels was 23.0 ± 4.5 months, but in the high BCL11A expression group, only 32.6% of patients relapsed at the endpoint of follow-up. The multivariate survival analysis indicated that BCL11A expression level was an independent marker of DFS in patients with NSCLC (hazards ratio [HR] 0.40, 95% confidence interval [CI] 0.22–0.72, *P* = 0.002). Furthermore, we found that in a subgroup of patients with early stage cancer (IA–IIB) (Table 2), and in particular early SCC (Table 3), BCL11A was not only a significant prognostic factor for DFS (HR 0.18, 95% CI 0.05–0.66, *P* = 0.01), but also for OS (HR 0.17, 95% CI 0.06–0.50, *P* = 0.001) (Figure 3C-F). No correlations between BCL11A expression and DFS or OS were observed in advanced stage (IIIA–IV) NSCLC patients (Additional file 1: Figure S1).

**Table 2:** Multivariate Cox regression analysis of BCL11A and survival in patients with early stage cancer (IA–IIB)

**Variable**	**Hazards ratio (95% CI)**	**Unfavorable / favorable**	***P *value**
**OS**			
Age	1.07 (1.03**–**1.12)	≥ 60/< 60	0.002
Tumor size	1.29 (1.08**–**1.57)	≥ 3 cm/< 3 cm	0.007
Lymph node status	9.09 (3.58**–**23.8)	Positive/negative	0.000
Performance status	2.46 (1.06**–**5.68)	1-2/0	0.036
BCL11A protein	0.27 (0.11**–**0.69)	Higher/lower	0.006
Histological type			0.003
SCC^#^	0.16 (0.05**–**0.54)	SCC/AC	0.003
LCC^※^	0.10 (0.03**–**0.38)	LCC/AC	0.001
**DFS**			
Tumor size	1.32 (1.11**–**1.56)	≥ 3 cm/< 3 cm	0.002
Lymph node status	2.56 (1.32**–**5.00)	Positive/negative	0.006
BCL11A protein	0.25 (0.12**–**0.51)	Higher/lower	0.001

^#^SCC *vs*. AC; ^※^LCC *vs*. AC.

**Table 3:** Multivariate Cox regression analysis of BCL11A and survival in patients with early stage squamous carcinoma

**Variable**	**Hazard ratio (95% CI)**	**Unfavorable / favorable**	***P *value**
**OS**			
Tumor size	1.38 (1.00**–**1.91)	≥ 3 cm/< 3 cm	0.049
BCL11A protein	0.18 (0.05**–**0.66)	Higher/lower	0.010
**DFS**			
BCL11A protein	0.17 (0.06**–**0.50)	Higher/lower	0.001

### *BCL11A* gene copy number gains in lung squamous carcinoma

**Figure 4:**
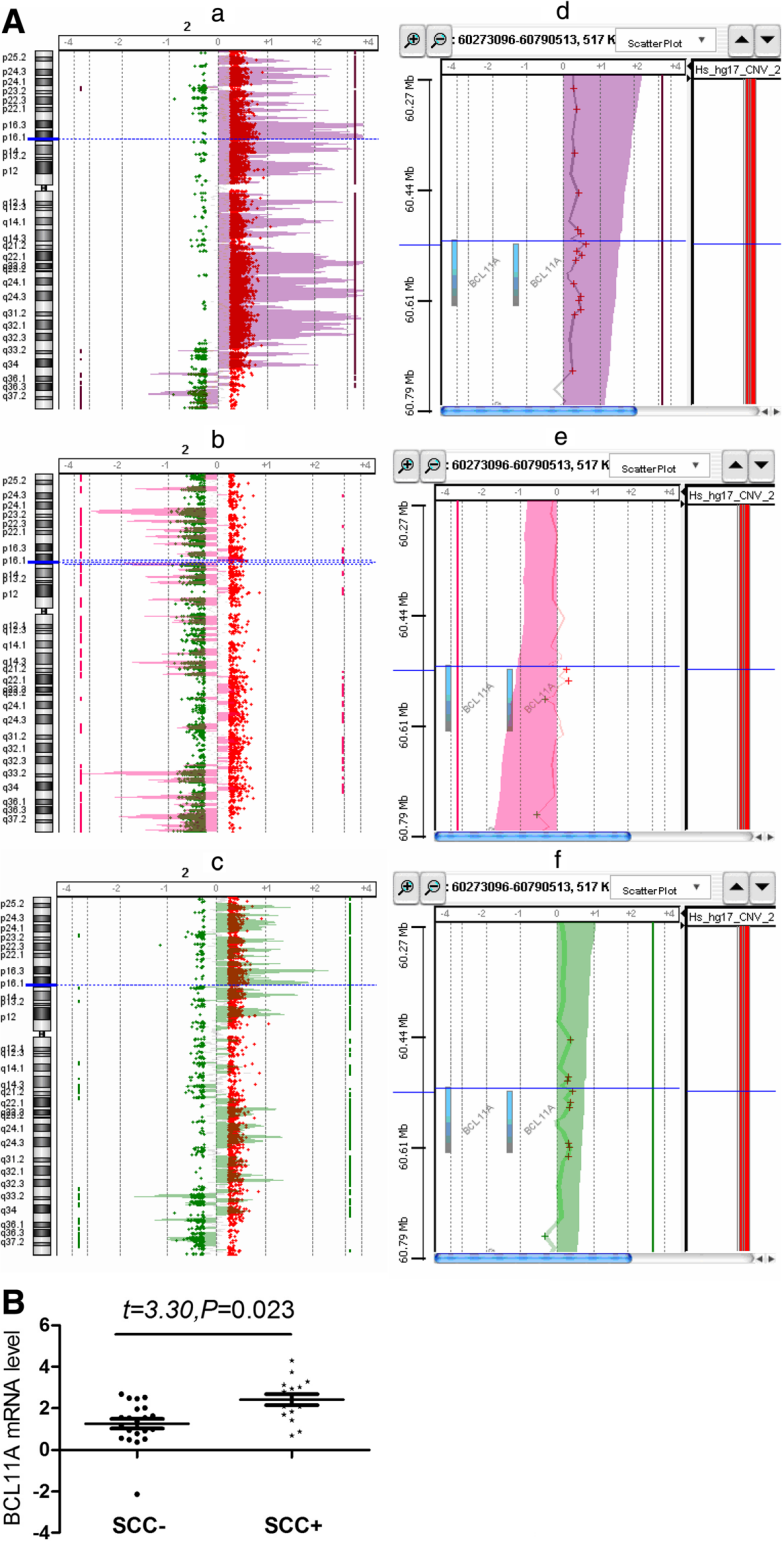
***BCL11A *gene amplification is frequently observed in squamous cell carcinoma.** (**A**) Chromosome and gene views of copy number variance (CNV) signals in NSCLC with different histological origins. Log2 ratios of CNV probe intensity are plotted on the *x* axis against genomic location on the *y* axis. The blue horizontal line represents the location of the *BCL11A* gene on chromosome 2. **a**, **b**, and **c** represent the chromosome views of squamous cell carcinoma, adenocarcinoma, and large cell carcinoma, respectively. **d**, **e**, and **f** represent the gene views of squamous cell carcinoma, adenocarcinoma, and large cell carcinoma, respectively, which show a zoom-in view of *BCL11A* copy number variation on the 2p16.1 band. (**B**) Expression levels were compared between SCC samples without high copy number amplification (SCC-) and SCC with high copy number amplification (SCC+) of the genomic region harboring the gene of interest.

Ten probes targeting *BCL11A* on the Agilent aCGH chip (A_16_P15674055, A_14_P129011, A_16_P15674243, A_16_P00386603, A_16_P00386649 and A_14_P106886) were used to determine the normalized logarithmic intensity of BCL11A copy number changes. The average intensity of the 10 probes for *BCL11A* in SCC was 0.35, significantly higher than that in adenocarcinoma (0.03) (*t* = 5.37, *P* = 0.0007). Log intensities >0.30 were considered indicative of gene amplification in aCGH analysis. Thus, *BCL11A* was amplified in SCC, while in large-cell carcinoma (LCC) (n = 11) the intensity was 0.23, indicating no *BCL11A* gene amplification (Figure 4A). A significantly higher average expression level was observed for *BCL11A* in the SCC samples with amplifications as compared to SCC samples without amplifications (t = 3.30, *P* = 0.023) (Figure 4B).

### Predicted microRNAs targeting BCL11A were downregulated in NSCLC tissues

**Figure 5:**
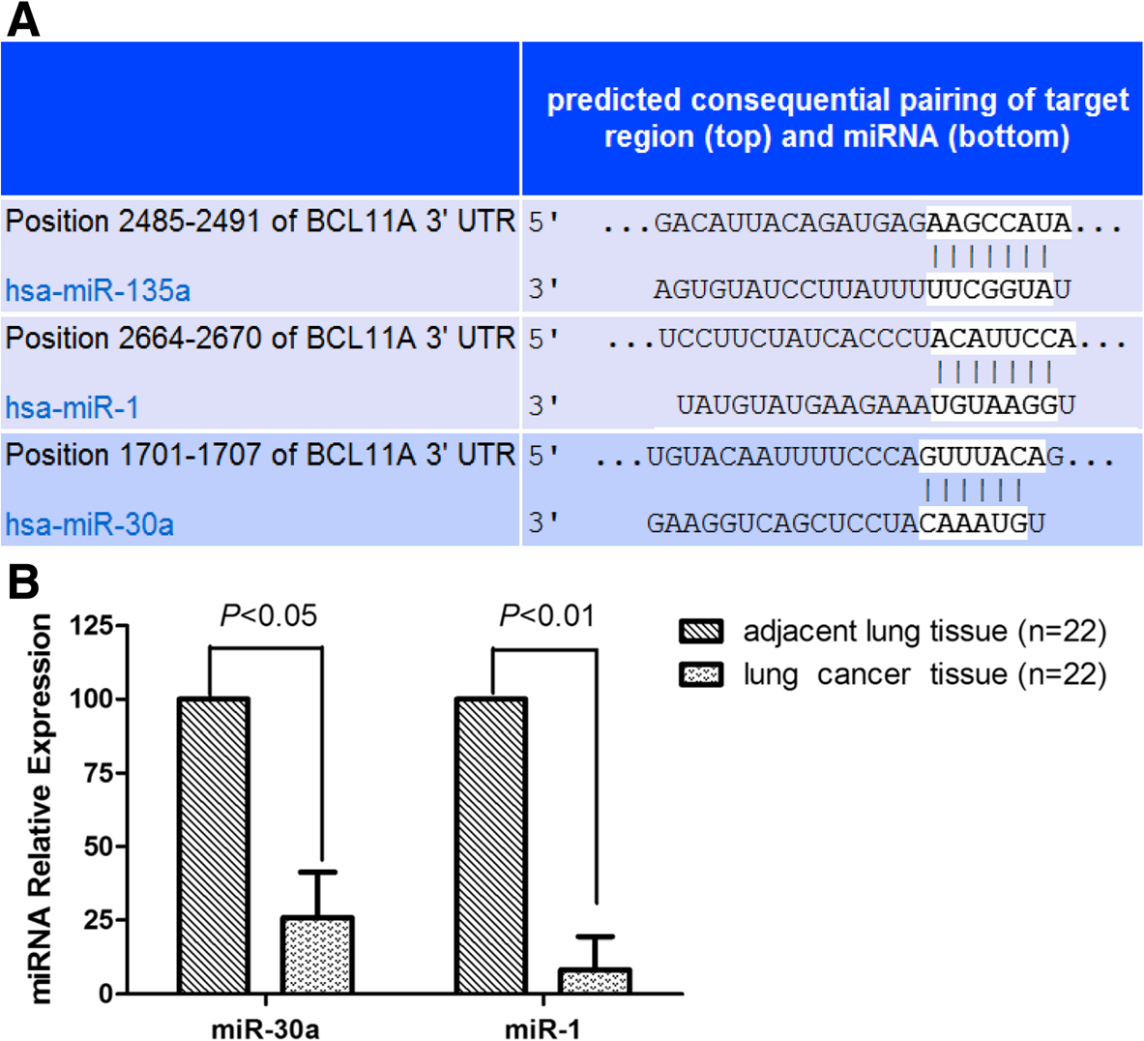
**Computationally predicted miRNAs targeting BCL11A are downregulated in NSCLC tissues.** (**A**) Schematic of three different binding sites in the *BCL11A* 3′UTR complementary to the “seed region” of three miRNAs, including miR-135a, miR-1, and miR-30a. (**B**) miR-1 and miR-30a were downregulated in NSCLC tissues compared to adjacent lung tissues (n = 22).

MicroRNAs (miRNAs) have gained considerable attention as regulators of gene expression [10], and they play important roles in cellular differentiation and embryonic stem cell development [11]. We postulated that they may also play a role in modulating BCL11A expression. In order to test whether miRNAs play a role in controlling BCL11A expression, we computationally identified those that might contribute to BCL11A regulation. Since it has been generally accepted that intersecting the results of multiple prediction algorithms can increase specificity, we chose to intersect the results of the prediction programs PicTar (4-way), miRanda (miRBase), and TargetScan [12]–[14]. Overall, only three miRNAs were found by these programs to target BCL11A, including miR-1, miR-135a, and miR-30a (Figure 5A). We intersected these three miRNAs with results of microarray-based miRNA expression profiles (data not shown) and found that only miR-1 and miR-30a were downregulated in NSCLC tissues. We further analyzed the expression of these two microRNAs in 22 paired tissues of NSCLC patients by TaqMan real-time PCR. As compared to adjacent tissues, miR-30a and miR-1 were downregulated by 3.9- and 12.1-fold in cancer tissues, respectively (Figure 5B).

### BCL11A is the target of miR-30a

**Figure 6:**
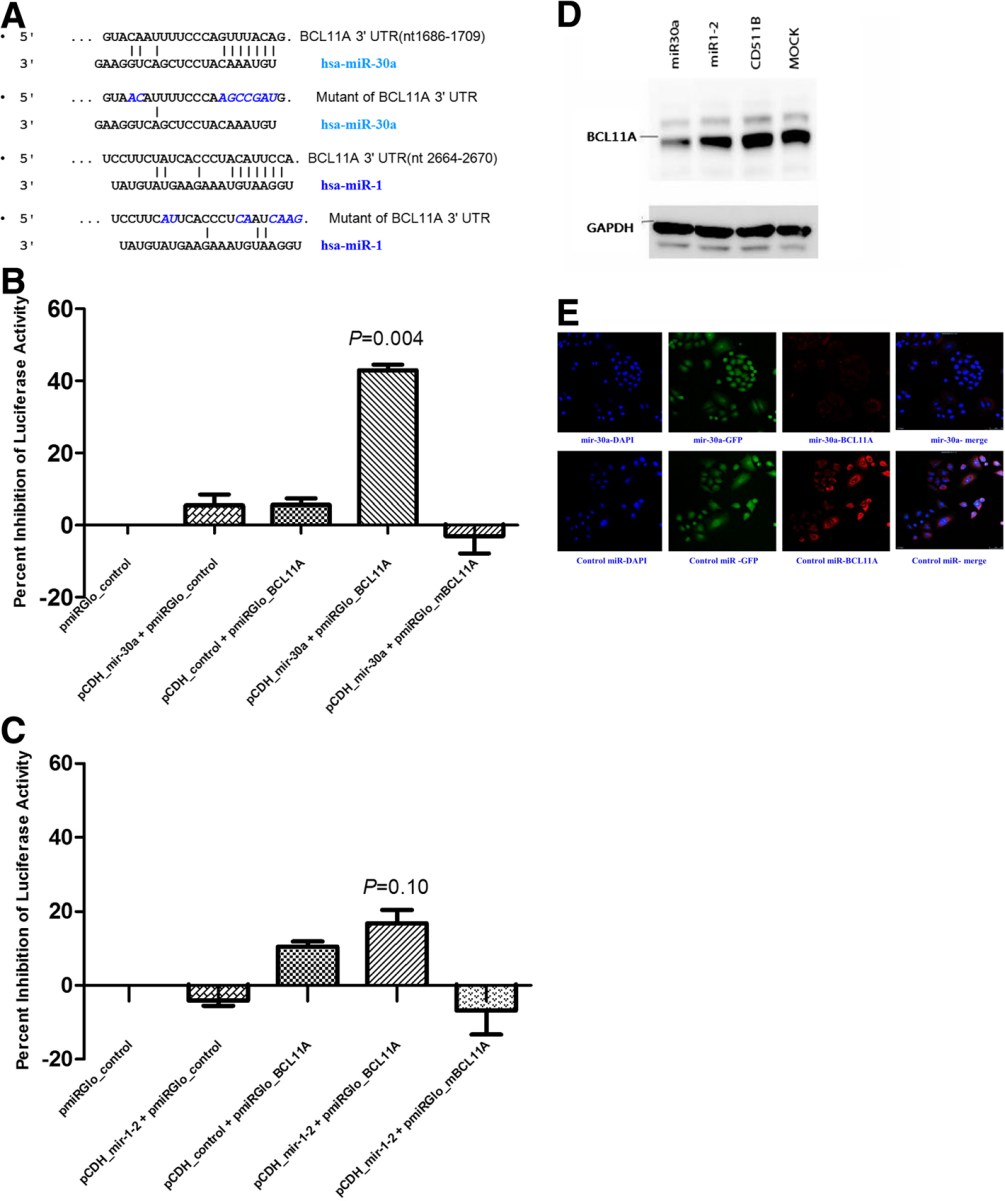
**The *BCL11A *3**′**-UTR is the target of miR-30a, but not of miR-1.** pmirGLO-*BCL11A* luciferase constructs, containing a wild-type or mutated *BCL11A* 3′-UTR (**A**), were transfected into A549 cells with (**B**) miR-30a, (**C**) miR-1, or control miRNA vectors. Relative repression of *firefly* luciferase expression was standardized to *Renilla* luciferase activity. The reporter assays were performed three times with essentially identical results. The *P* value was calculated comparing control microRNA and miR-30a or miR-1. (**D**) miR-30a downregulated BCL11A protein expression in H520 cells. Control miRNAs were also used for transfection. The experiment was performed three independent times, and representative results are displayed. CD511B represents the pCDH-CMV-MCS-EF1-copGFP empty vector. (**E**) Immunofluorescence staining intensity of BCL11A protein in H460 cells observed by confocal microscopy after transfection with miR-30a or control miRNA. Magnification, ×200.

After co-transfection of pmirGLO dual luciferase reporter plasmids carrying the 3′-UTR sequence of BCL11A and plasmids expressing miR-30a, miR-1, or a control miRNA into A549 and NIH3T3 cells, *firefly* luciferase activity was normalized to *Renilla* luciferase activity. Significant repression was only seen in miR-30a-transfected cells, but not in either miR-1- or control miRNA-transfected cells. When the nucleotide acid sequence in the BCL11A 3′UTR that was complementary to the “seed region” of miR-30a was replaced, the repression effect was completely rescued (Figure 6A-C).

### BCL11A protein expression is inhibited by miR-30a *in vitro*

Since the 3′UTR of BCL11A could be a target of miR-30a, we tested whether 3′UTR binding by miR-30a resulted in changes in BCL11A protein expression in H460 and H520 cell lines. First, we performed an immunoblot analysis using a BCL11A-specific antibody. After transfection with miRNAs, BCL11A protein expression in the H520 cell line was repressed by miR-30a, but not by miR-1 or control miRNA (Figure 6D). After transient transfection with a plasmid expressing miR-30a, BCL11A staining in H460 cells was significantly downregulated compared to those transfected with control miRNA, as determined by immunofluorescence and confocal microscopy (Figure 6E).

## Discussion

The current study investigated the role of the proto-oncogene *BCL11A* (B-cell lymphoma/leukemia 11A) in NSCLC. Our results demonstrated that BCL11A was upregulated in clinical NSCLC tissue samples at both transcriptional and translational levels. Statistical analyses demonstrated that BCL11A expression was associated strongly with lymph node status, disease stage, and the survival of patients with NSCLC. Moreover, we also observed some significant differences between BCL11A protein expression and histologic origin. The survival analysis found that patients with higher BCL11A expression had better outcomes. BCL11A protein was an independent prognostic factor of disease-free survival and overall survival for early stage patients (IA–IIB), especially for early stage squamous carcinoma patients.

The proto-oncogene *BCL11A* spans over 102 kb on chromosome 2p16 and is implicated in several neoplasms [9,15]. Its sequence is phylogenetically conserved and codes for a zinc-finger protein with an unusual C_2_HC zinc finger at the N-terminus and six other Krüppel-like C_2_H_2_ zinc fingers near the C-terminus [10]. BCL11A was reported to show restricted expression in fetal liver, bone marrow, lymphoid, and brain tissue [16,17], but our results clearly demonstrated that the proto-oncogene *BCL11A* was actively expressed in NSCLC. This phenomenon suggested that abnormal activation of the proto-oncogene *BCL11A* might contribute to tumorigenesis of NSCLC, although the details of its function have not yet to be clarified. BCL11A can bind to 5′-GGCCGG-3′ DNA motifs with high affinity, and can promote the deacetylation of histone H3/H4 associated with the promoter reigons of mammalian target genes, thereby causing transcriptional repression [15,18]. A previous study reported that enhanced BCL11A expression could repress P21 induction, which correlated with reduced colony formation and cell cycle arrest of leukemic cells [19]. Yin *et al*. also reported that *BCL11A* acts as an oncogene and causes leukemia in the absence of *NF1* in mice, perhaps through suppression of P21 induction and thus promotion of cell growth [20]. As is well known, the *NF1* suppressor gene is also frequently inactivated in NSCLC [21]. Whether the same event occurs in NSCLC tumorigenesis remains unclear. Apart from transcriptional repression, Yu *et al*. found that BCL11A can also upregulate expression of BCL2, BCL2-xL, and MDM2 which inhibits P53 activities [22]. These molecular alterations occur frequently in solid tumors including lung cancer. Therefore, BCL11A may contribute to tumorigenesis through effects on apoptosis, the cell cycle, and DNA damage repair, but the exact mechanisms warrant further investigation.

When compared to patients with advanced stage NSCLC and lymph node involvement, BCL11A expression was much higher in patients with early stage cancer and no lymph node involvement, which suggests that activation of the *BCL11A* proto-oncogene might be an early stage event in NSCLC. We performed a survival analysis and found that high BCL11A expression correlated with better survival and DFS, results similar to previous data on lymphoma. Pulford *et al*. performed immunolabelling studies on 107 cases of DLBCL (diffuse large B-cell lymphoma) and showed a trend towards poorer survival in patients with BCL11A-xL-negative tumors [23]. Recently, it was reported that *BCL11A* was amplified in lung squamous cell carcinoma (SCC), and this amplification was significantly more frequent in SCC samples of NSCLC patients without metastases [24]. In our study, BCL11A protein level was a protective factor for both DFS and OS of early stage NSCLC patients, especially for early stage SCC. Therefore, one wonders whether BCL11A inhibits cancer metastasis and acts as a tumor suppressor. Liu *et al*. found that transplantation of *BCL11A*-knockout murine fetal liver cells resulted in T-cell leukemia in recipient mice, suggesting that *BCL11A* may be a non–cell autonomous T-cell tumor suppressor gene [6]. Moreover, another member of the BCL11 family, *BCL11B*, has been suggested as a tumor suppressor gene, acting via an increase in resistance to DNA damage [25,26]. In addition to the potential mechanism mentioned above, two cell cycle checkpoint members, P21 and CHEK1, which contribute to several cell cycle checkpoints, including the G1/S and G2/M checkpoints might also account for this process. Activation of P21 and CHEK1 can promote DNA repair, maintain genomic integrity, and attenuate chemo- or radiotherapeutic efficacy [27,28]. Collective evidence suggests that P21 and CHEK1 could be targets of BCL11A. This interesting phenomenon implied BCL11A might have a dual role during NSCLC progression. In the initiation of tumorigenesis, BCL11A might act as an oncogene by repressing P21, CHEK1, and P53, resulting in genomic instability and contributing to carcinogenesis. However, once the tumor is established, BCL11A might inhibit cancer metastasis and improve the sensitivity of chemo- or radiotherapy, thereby contributing to better survival. This hypothesis should be further investigated in future studies.

There were several mechanisms that accounted for the abnormal activation of the proto-oncogene *BCL11A* reported in malignant hematological disease, including genomic amplification, chromosomal translocation, and retroviral integration. In this study, *BCL11A* gene copy number gains were frequently observed in squamous cell lung cancer patients, but not in adenocarcinoma or large cell carcinoma patients, which matched the results of Boelens *et al*. They observed that about 12% of squamous cell lung cancers had high copy number amplifications on chromosomal regions 2p15–p16.1, In addition, a significantly higher average expression level was observed for *BCL11A* in the SCC samples with amplifications as compared to SCC without amplifications. Combined results demonstrated that genomic amplification is partially responsible for abnormal BCL11A expression in SCC tissues. MicroRNAs are approximately 21-nucleotide-long RNA regulators of gene expression at the translational level and control gene expression post-transcriptionally by regulating mRNA translation in the cytoplasm [29,30]. Using the miRGen database [14], we found that BCL11A is a common target of miR-1 and miR-30a, which were downregulated in NSCLC compared to normal lung tissues. Thus, we performed miRNA transfection to explore whether downregulation of miRNAs in NSCLC tissues contributed to deregulation of BCL11A. The results demonstrated that miR-30a could inhibit BCL11A protein expression *in vitro.* Thus, inactivation of miR-30a might also contribute to proto-oncogene *BCL11A* activation. However, whether these two mechanisms act synergistically remains unknown. In addition, we did not elucidate the biological functions of BCL11A and miR-30a in NSCLC.

In summary, we report here for the first time the potential role of BCL11A in diagnosing and predicting the prognosis of patients with NSCLC, especially those with early stage lung squamous carcinoma. BCL11A was highly and specifically expressed in cancer tissues rather than adjacent non-cancerous tissues. Deregulated expression of BCL11A might be partly caused by miR-30a inactivation and genomic *BCL11A* amplification. Thus, future studies should assess whether *BCL11A* functions as an oncogene or tumor suppressor gene in NSCLC, whether BCL11A can be a better diagnostic and prognostic biomarker of early stage squamous cell lung cancer.

## Conclusions

Activation of the proto-oncogene *BCL11A* may be a potential diagnostic and prognostic biomarker of NSCLC, and is modulated by miR-30a and gene amplification.

## Methods

### Patients and tissue samples

Surgical specimens were obtained with informed consent from 114 NSCLC patients (81 males and 33 females) who underwent potentially curative surgery at Guangdong General Hospital between 2003 and 2006. This study was approved by the Institutional Review Board (IRB) of Guangdong General Hospital. The staging and histological classifications were based on the World Health Organization (WHO) system. A follow-up evaluation was performed according to standard follow-up protocol. The median follow-up period was 47.2 months (range, 3.1–75.2 months). Hemotoxylin and eosin (H&E) staining was performed on sections of each tissue to determine the percentage of tumor cells by two independent pathologists. Only those samples with tumor content ≥ 80% were allowed to enter this study. Demographic and clinical characteristics of the patients are listed in Additional file 2: Table S1.

### Cell lines

The NIH3T3, A549,NCI-H460 and H520 cell lines were obtained from the American Type Culture Collection (Manassas, VA, USA) and were cultured in RPMI 1640 medium (Invitrogen, Carlsbad, CA) supplemented with 10% fetal bovine serum (Thermo, Waltham, MA). Cells were maintained at 37°C in a humidified 5% CO_2_ incubator (Thermo).

### Gene expression profiling using Affymetrix GeneChip® technology

Cancer tissue samples from 114 cases and 53 adjacent non-tumor tissue samples were subject to gene profiling tests using the Affymetrix human genome U133 plus 2.0 GeneChip® (Affymetrix, Santa Clara, CA, USA). Agilent GeneSpring® software (Agilent Technologies, Santa Clara, CA, USA) was utilized for the analysis of differentially expressed genes between cancerous and adjacent non-cancerous tissues with a Bonferroni correction FDR (false discovery rate) of 0.0001. Normalized intensities of Affymetrix probe sets targeting *BCL11A* (222891_s_at, 219497_s_at, 219498_s_at, 1559078_s_at, and 210347_s_at) were extracted and averaged for plotting across all samples.

### Immunohistochemistry on tissue microarrays

Tissue microarrays were constructed using a manually operated tissue arrayer (Beecher Instruments, Sun Prairie, WI, USA). A total of 113 formaldehyde-fixed paraffin-embedded (FFPE) samples were successfully used to generate four serial tissue arrays. Immunohistochemical staining process was performed according to the protocol provided by DAKO (DakoCytomation, Glostrup, Denmark) [31]. A primary mouse monoclonal antibody against human BCL11A (ab19487; Abcam, Cambridge, USA) was applied to the sections at a dilution of 1:200 for 1 h at room temperature. The sections were counterstained with Harris’s hematoxylin. Each tumor was assigned a score according to the intensity of the nucleic or cytoplasmic staining (0 = no staining , 1 = weak staining, 2 = moderate staining, and 3 = strong staining) and the proportion of stained tumor cells (0 = 0%, 1 = 1–10%, 2 = 11–50% , 3 = 51–80%, and 4 = 81–100%) [31], as judged by two pathologists, independently. The final immunoreactive score was determined by multiplying the intensity scores by the extent of positivity scores of stained cells, with a minimum score of 0 and a maximum score of 12. Tumors with scores ≥ 6 were classified into the high BCL11A expression group, while the others were classified into the low BCL11A expression group.

### Array-based comparative genome hybridization (aCGH) analysis

Array-CGH tests were performed using the Agilent Human Genome Microarray kit 244A (Agilent Technologies). Labeling and hybridization were performed according to the protocol provided by Agilent (Protocol v4.0, June 2006). Ten probes targeting BCL11A on the Agilent aCGH chip (A_16_P15674055, A_16_P15674092, A_16_P00386506, A_14_P202460, A_16_P15674199, A_14_P129011, A_16_P15674243, A_16_P00386603, A_16_P00386649, and A_14_P106886) were used to determine the normalized logarithmic intensity of *BCL11A* copy number changes. Log intensities > 0.30 were considered to indicate gene amplification.

### BCL11A-targeting microRNA prediction

To identify miRNAs targeting BCL11A, we integrated the output results from the DIANA miRGen online database, which were generated using multiple prediction programs: TargetScan [http://www.targetscan.org/], PicTar [http://www.pictar.org/], miRanda [http://www.microrna.org/microrna/] and DIANA microT [http://diana.pcbi.upenn.edu/cgi-bin/micro_t.cgi] [14]. We integrated the results for each prediction tool and exported the sequences of predicted binding sites for each microRNA in the *BCL11A* 3′UTR.

### Quantification of microRNA expression using real time qRT-PCR

Twenty-two paired NSCLC tissues undergoing microRNA analyses included (nine cases of adenocarcinoma, eight of squamous cell carcinoma, and five of large cell carcinoma) were subjected to microRNA analyses. Quantitative RT-PCR amplification of microRNA and *RNU6* was performed using the pre-developed Assay-on-Demand Gene Expression Set for the microRNA (Applied Biosystems, Foster City, CA). Briefly, 10 ng of total RNA was reverse transcribed into cDNA using High Capacity cDNA sysnthesis kit (Applied Biosystems) in the presence of stem-loop primers. All reactions were performed in triplicates using 20 μl samples containing 1 ng of cDNA. The qRT-PCR reaction was performed using in an ABI PRISM 7500FAST system (Applied Biosystems), with the following cycling conditions: 10 min at 95°C, and 40 cycles of 95°C for 15 s and 60°C for 60 s. microRNA expression was quantified using the comparative CT (cycle at threshold) method, which normalizes the CT values to an internal housekeeping ncRNA (*RNU6*) and calculates the relative expression value [32].

### MicroRNA vectors and luciferase reporter assays

Two microRNA-expressing plasmids were constructed. DNA fragments carrying pre-microRNAs were amplified by high-fidelity PCR using genomic DNA from a healthy blood donor as a template. PCR reactions were performed using the high-fidelity Phusion polymerase (New England Biolabs), and specific primers ending with sequences for *Eco*RI and *Not*I enzymes (New England Biolabs) (Additional file 3: Table S2). The amplified fragment was enzyme digested and subsequently cloned into pCDH-CMV-MCS-EF1-copGFP (System Biosciences, Mountain View, CA) at the *Eco*RI and *Not*I sites. Expression of the mature microRNAs was verified by TaqMan real-time qRT-PCR. Luciferase constructs were created by ligating oligonucleotides (Additional file 4: Table S3) containing the wild type or mutant target site of the BCL11A 3-UTR into the *Dra*I and *Xba*I sites of the pmirGLO vector (Promega, Madison, WI). Vectors were sequenced using an *EBV* reverse primer: 5′-GTGGTTTGTCCAAACTCATC-3′. A549 and NIH3T3 cells (low endogenous BCL11A expression; data not shown) were co-transfected with 0.5 μg of pmirGLO luciferase reporter vector containing a wild type or mutant target site and 0.5 μg of the microRNA plasmid using Lipofectamine™ LTX reagent (Invitrogen, Carlsbad) in 12-well plates. After incubation for 48 h, the cells were lysed and luciferase assays were conducted using a GloMax™ 20/20 Luminometer (Promega). *Firefly* luciferase activity was normalized to *Renilla* luciferase activity. Each experiment was performed in triplicate [32].

### Western blotting

H520 cells (high endogenous BCL11A expression; data not shown) were transfected with the control microRNA- or miR-30a-expressing vector. Approximately 48 h after transfection, the cells were lysed. Western blotting was performed as described previously [33]. The anti-BCL11A antibody (ab19487) was obtained from Abcam and was used at a dilution of 1:1,000.

### Visualization of intracellular BCL11A protein expression by confocal microscopy

Indirect immunofluorescence staining was used to determine BCL11A expression in miR-30a-transfected cells as described previously [34]. Briefly, H460 cells (high endogenous BCL11A expression; data not shown) were transfected with the control microRNA- or miR-30a-expressing vector, and 48 h after transfection, the cells were fixed with cold methanol. A primary antibody against BCL11A (ab19487; Abcam) was applied at a dilution of 1:100, followed by a secondary antibody conjugated to Alexa Fluor 635 (Invitrogen). DAPI (Invitrogen) was used as a nuclear stain, and GFP was used to monitor transfection efficiency.

### Statistical analysis

Statistical significance was determined by paired or unpaired Student’s *t*-tests in cases of standardized expression data. One-way analysis of variance (ANOVA) was performed for comparisons of multiple groups using the GraphPad Prism Software 5.01 (GraphPad, La Jolla, CA). Wilcoxon matched-pair tests and Mann-Whitney U tests were used for nonparametric analysis of non-Gaussian data. Error bars represent standard deviations (± SD). Chi-square tests and McNemar’s tests were used to assess the association of BCL11A level with clinical variables. Survival curves between subgroups divided according to BCL11A expression level were drawn using the Kaplan-Meier method, and significant differences among subgroups were compared by log-rank test. A multivariate analysis was performed using the stepwise method. Hazards ratios and 95% confidence intervals were calculated using Cox proportional hazards models. *P* values < 0.05 were considered statistically significant.

## Competing interests

The author(s) indicated no potential conflicts of interest.

## Authors' contributions

BJ carried out the microRNA studies, data analysis and drafted the manuscript. XZ performed the molecular genetic studies and participated in the manuscript writing. XY, JY, QZ, ZC provided study material or patients and assembled data. JS, WM, ZC, ZX, SC carried out vector construction, real-time PCR,and immunohistochemistry. YW conceived the study, participated in its design, and coordination and helped to draft the manuscript. All authors read and approved the final manuscript.

## Supplementary Material

Additional file 1: Figure S1 Kaplan-Meier estimated overall survival (OS) and disease-free survival (DFS) curves of advanced NSCLC patients according to BCL11A protein level. Click here for file

Additional file 2: Table S1 Summary of the clinicopathological characteristics of the patients. Click here for file

Additional file 3: Table S2 Primers utilized for the cloning the microRNA expression vector. Click here for file

Additional file 4: Table S3` Wild type and mismatch *BCL11A* 3' UTR sequences ligated into pmirGLO vector. Click here for file

## Note

The English in this document has been checked by at least two professional editors, both native speakers of English. For a certificate, please see: http://www.textcheck.com/certificate/index/qhP4zS
