# Supplementary material for: BCL11A overexpression predicts survival and relapse in non-small cell lung cancer and is modulated by microRNA-30a and gene amplification
Source: Mol Cancer. 2013 Jun 12;12:61. doi: 10.1186/1476-4598-12-61 (PMC3695801; doi:10.1186/1476-4598-12-61)
Supplement: Additional file 1: Figure S1 — Kaplan-Meier estimated overall survival (OS) and disease-free survival (DFS) curves of advanced NSCLC patients according to BCL11A protein level. [file 1476-4598-12-61-S1.docx]

**Figure.S1**

**Supplemental Figure S1. Kaplan-Meier estimated overall survival (OS) and disease-free survival (DFS) curves of advanced NSCLC patients according to BCL11A protein level.**

(A). Disease-free survival of 25 advanced stage NSCLC patients

(B). Overall survival of 25 advanced stage NSCLC patients

**(A) Advanced 25: BCL11A protein vs DFS**

**(B) Advanced 25: BCL11A protein vs OS**
