# Supplementary material for: BCL11A overexpression predicts survival and relapse in non-small cell lung cancer and is modulated by microRNA-30a and gene amplification
Source: Mol Cancer. 2013 Jun 12;12:61. doi: 10.1186/1476-4598-12-61 (PMC3695801; doi:10.1186/1476-4598-12-61)
Supplement: Additional file 2: Table S1 — Summary of the clinicopathological characteristics of the patients. [file 1476-4598-12-61-S2.docx]

**Table. s1. Summary of the clinicopathological characteristics of the patients.**

| Characteristic | Number (%) |
| --- | --- |
| No. of patients | 114 |
| Age (years) (mean ± S.D.) | 60.8 ± 10.1 |
| < 60 | 50 (43.9%) |
| ≥ 60 | 64 (56.1%) |
| Sex |  |
| Male | 81 (71.1%) |
| Female | 33 (28.9%) |
| Smoking status |  |
| Smoker | 61 (53.5%) |
| Non-smoker | 53 (46.5%) |
| Histology |  |
| Adenocarcinoma (AC) | 68 (59.6%) |
| Squamous cell carcinoma (SCC) | 35 (30.7%) |
| Large-cell carcinoma (LCC) | 11 (9.6%) |
| Stage |  |
| I | 69 (60.5%) |
| II | 20 (17.5%) |
| III | 23 (20.2%) |
| IV | 2 (1.8%) |
