# Supplementary material for: BCL11A overexpression predicts survival and relapse in non-small cell lung cancer and is modulated by microRNA-30a and gene amplification
Source: Mol Cancer. 2013 Jun 12;12:61. doi: 10.1186/1476-4598-12-61 (PMC3695801; doi:10.1186/1476-4598-12-61)
Supplement: Additional file 3: Table S2 — Primers utilized for the cloning the microRNA expression vector. [file 1476-4598-12-61-S3.doc]

Table. s2. Primers utilized for the cloning the microRNA expression vector.

| Gene | Primer Sequence |
| --- | --- |
| mir-1-2 | F 5’-***CGGAATTCCG***AATTGCTCTACATTAGTAAGC -3’  R 5’-***ATAAGAATGCGGCCGCTAAACTAT***TAGTATTGCCAAAGGTCATC -3’ |
| mir-30a | F 5’- ***CGGAATTCCG***GTGGCTAACAATAATGAATGAAACC-3’  R 5’-***ATAAGAATGCGGCCGCTAAACTAT***ATGCCCTGCTGAAGCCCTCTAA-3’ |

Italic bold capitals represent the [restriction endonuclease recognition sequences](http://www.jstor.org/stable/10.2307/2408332) and conservative base sequences.
