# Supplementary material for: BCL11A overexpression predicts survival and relapse in non-small cell lung cancer and is modulated by microRNA-30a and gene amplification
Source: Mol Cancer. 2013 Jun 12;12:61. doi: 10.1186/1476-4598-12-61 (PMC3695801; doi:10.1186/1476-4598-12-61)
Supplement: Additional file 4: Table S3` — Wild type and mismatch BCL11A 3' UTR sequences ligated into pmirGLO vector. [file 1476-4598-12-61-S4.docx]

Table. s3. Wild type and mismatch *BCL11A* 3' UTR sequences ligated into pmirGLO vector.

| Target | Oligonucleotide sequences |
| --- | --- |
| miR-30a | BCL11A-*Dra*I-F:  *AAAC* TA *GCGGCCGC* TAGT **ACAATTTTCCCAGTTTACAG** *T*  BCL11A-*Xba*I-R:  *CTAGA* **CTGTAAACTGGGAAAATTGT** ACTA *GCGGCCGC* TA *GTTT*  mut_BCL11A-*Dra*I-F:  *AAAC* TA *GCGGCCGC* TAGT **AACATTTTCCCAAGCCGATG** *T*  mut_BCL11A-*Xba*I-R:  *CTAGA* **CATCGGCTTGGGAAAATGTT** ACTA GCGGCCGC TA *GTTT* |
| miR-1 | BCL11A-*Dra*I-F:  *AAAC* TA *GCGGCCGC* TAGT **CCTTCTATCACCCTACATTCCA** *T*  BCL11A-*Xba*I-R:  *CTAGA* **TGGAATGTAGGGTGATAGAAGG** ACTA *GCGGCCGC* TA *GTTT*  mut_BCL11A_*Dra*I-F:  *AAAC* TA *GCGGCCGC* TAGT **CCTTCATTCACCCTCAATCAAG** *T*  mut_BCL11A-*Xba*I-R:  *CTAGA* **CTTGATTGAGGGTGAATGAAGG** ACTA *GCGGCCGC* TA *GTTT* |

Constructs contained either an exact match to the miR-30a and miR-1 target sequence or a mismatched version of that target site is in bold, the [restriction endonuclease cleavage site](http://www.jstor.org/stable/10.2307/2408332) is in italics.
